# Genetic Analysis of East Asian Grape Cultivars Suggests Hybridization with Wild *Vitis*

**DOI:** 10.1371/journal.pone.0140841

**Published:** 2015-10-21

**Authors:** Nami Goto-Yamamoto, Jason Sawler, Sean Myles

**Affiliations:** 1 National Research Institute of Brewing, Higashi-Hiroshima, Japan; 2 Faculty of Agriculture, Dalhousie University, Truro, Nova Scotia, Canada; 3 Anandia Labs, 2259 Lower Mall, Vancouver, British Columbia, Canada; Univeristy of California Davis, UNITED STATES

## Abstract

Koshu is a grape cultivar native to Japan and is one of the country’s most important cultivars for wine making. Koshu and other oriental grape cultivars are widely believed to belong to the European domesticated grape species *Vitis vinifera*. To verify the domesticated origin of Koshu and four other cultivars widely grown in China and Japan, we genotyped 48 ancestry informative single nucleotide polymorphisms (SNPs) and estimated wild and domesticated ancestry proportions. Our principal components analysis (PCA) based ancestry estimation revealed that Koshu is 70% *V*. *vinifera*, and that the remaining 30% of its ancestry is most likely derived from wild East Asian *Vitis* species. Partial sequencing of chloroplast DNA suggests that Koshu’s maternal line is derived from the Chinese wild species *V*. *davidii* or a closely related species. Our results suggest that many traditional East Asian grape cultivars such as Koshu were generated from hybridization events with wild grape species.

## Introduction

Grapes are one of the most important horticultural crops worldwide, and are typically consumed as fresh fruit or used in the production of wine, brandy, juice and raisins. The majority of wine grapes are cultivars of the domesticated grape *V*. *vinifera*, which originated in the Caucasus and spread from there to Europe and eventually to grape growing regions worldwide [1]. Cultivars grown for sale as table grapes or juice are often interspecific hybrids of *V*. *vinifera* and wild species (e.g. *V*. *labrusca*). There are an estimated 60 species in the genus *Vitis* distributed broadly across the entire northern hemisphere, and many wild grapevines are used to breed new cultivars since they harbor desirable traits like disease resistance and tolerance to abiotic stress [2].

Several grape cultivars from China and Japan are widely grown for commercial purposes. For example, Koshu is one of Japan’s most widely planted and popular grape cultivars with approximately 1200 acres under vine in 1997 [3]. It is known for its distinct pale purple skin containing both cyanidin-based and delphinidin-based anthocyanins [4], but not anthocyanin diglucosides [5]. Wine produced from Koshu has aromatic characteristics similar to Sauvignon blanc [6] and has contributed significantly to the growth of the Japanese wine industry.

It is widely believed that Koshu is an oriental cultivar of *V*. *vinifera* [3] and previous analyses have supported this hypothesis. For example, a phylogeny generated using simple sequence repeats (SSRs) showed that Koshu clusters with well-known *V*. *vinifera* cultivars such as Sultanina and Muscat of Alexandria [7]. Similar results were observed from analyses of AFLPs [8] and principal coordinate analysis of SSR data [9]. In contrast, there is also evidence that Koshu may have ancestry from wild *Vitis* species. For example, Koshu has the SSR allele 4MG1 or n+4 at the VVS2 locus [10], which has only been found in rootstock cultivars, wild species, or cultivars crossed with wild *Vitis* (Boursiquot JM, personal communication). Similarly, Koshu and other East Asian cultivars possess unique SSR alleles that are not found in European *V*. *vinifera* cultivars [9, 10].

To verify the ancestry of popular East Asian cultivars on a genome-wide scale, we genotyped a panel of 48 ancestry informative markers (AIMs) in the Japanese cultivars Koshu and Koshu-sanjaku, and the Chinese cultivars Longan, Huotianhong, Baijixin. The AIMs are a subset of SNPs from the Vitis9kSNP array [11] that clearly differentiate wild *Vitis* species from *V*. *vinifera*. Our ancestry estimates are based on a method developed by Sawler et al. [12], who showed that genotyping fewer than 50 SNPs leads to accurate estimates of the genomic contributions of *V*. *vinifera* and North American wild *Vitis* in interspecific hybrid grape cultivars from the USDA grape germplasm collection. Here we apply the same method to quantify the potential genomic contribution of wild East-Asian *Vitis* to popular East Asian cultivars. In addition, the maternal ancestry of these cultivars was investigated by partially sequencing their chloroplast DNA and comparing them to published sequences.

## Materials and Methods

### Marker selection and genotyping

Forty-eight AIMs were selected as those with the highest loadings along the first principal component (PC1) from PCA performed on 6114 SNPs genotyped in 1031 samples labeled as *V*. *vinifera* and 786 labeled as wild *Vitis* grapevine accessions from the USDA grape germplasm collection [12]. For each SNP, pairs of PCR primers were designed based on 100 bp flanking each side of the SNP. The amplified fragments were purified with a Nucleospin Gel and PCR Clean-up kit (Macherey-Nagel, Düren, Germany) and sequenced directly, or after gel electrophoresis and extraction from the gel when necessary. The genotyped SNPs and their primer sequences according to the 8x Pinot Noir reference genome [13] are shown in S1 Table.

### Genetic material

The ten DNA samples genotyped for the AIMs in this study are Koshu, Pinot Noir, Koshu-sanjaku, Longan, Huotianhong, Baijixin, *V*. *amurensis*, *V*. *coignetiae*, *V*. *ficifolia* var. ganebu, and *V*. *shiragai*. The full genotype data are shown in S2 Table. Among them *V*. *amurensis*, *V*. *coignetiae*, *V*. *ficifolia* var. ganebu, and *V*. *shiragai* were extracted using standard methods from young leaves donated by Prof. Horiuchi and Dr. Mochioka of Osaka Prefecture University. All other DNA was extracted from young leaves of vines in an experimental vineyard in Higashi-Hiroshima.

For the analysis of chloroplast DNA, partial chloroplast sequences were obtained from the following six cultivars: Koshu, Koshu-sanjaku, Longan, Huotianhong, Baijixin, and Chardonnay.

### Principal components analysis and ancestry estimation

PCA of the 48 AIMs was first performed using 33 *V*. *vinifera* cultivars and 33 accessions of East-Asian wild *Vitis* species. These two groups of accessions are considered ancestral populations for the purposes of this study. Except for four East Asian wild *Vitis* samples obtained for this study, the genotypes for these ancestral groups were obtained from previously published data from the Vitis9KSNP genotyping microarray [14, 15]. Genotype data from the 48 AIMs were obtained for this study by genotyping four East Asian wild species (*V*. *amurensis*, *V*. *coignetiae*, *V*. *ficifolia var*. *ganebu*, and *V*. *shiragai*), five oriental cultivars (Koshu, Koshu–sanajku, Longan, Huotianhong, Baijixin) and one *V*. *vinifera* (Pinot noir). After establishing the PC axes using the 33 *V*. *vinifera* and 33 wild accessions described above as ancestral populations, the remaining samples were subsequently projected onto the first two PCs. The proportion of *V*. *vinifera* ancestry in the samples genotyped as part of this study was then calculated as follows: ‘% *V*. *vinifera* = b/(a+b)’, where a and b are the chord distances along the first principal component from the centroids of the *V*. *vinifera* cultivars and wild species in PC space, respectively, according to [12].

### Partial sequencing of chloroplast DNA

Following the method of Zecca et al. [16] with minor modification, four spacer regions of chloroplast DNA (*trnH*-*psbA*, *trnK*-*rps16*, *trnF*-*ndhJ*, and *rpl32*-*trnL*) were amplified and sequenced. The PCR and sequencing primers are shown in S3 Table. The sequences obtained from this have been submitted to DDBJ [LC054004—LC054031].

The reported sequences of wild *Vitis* species and those of *V*. *vinifera* subsp. *sylvestris* (HQ656029-HQ656577) were downloaded from GenBank and compared with the sequence obtained in this study.

### Phylogenetic analysis of chloroplast sequence

The wild species for which sequence data were unobtainable from one or more of the four chloroplast genomic regions were omitted from the analysis. The downloaded sequences were trimmed to the same region of the sequence obtained in this study after alignment with ClustalW. The total resulting sequence length was 2081 bp including gaps. For each accession, the four regions were joined and a dendogram was constructed using the Neighbor-Joining (NJ) method in MEGA6 [17]. Distances were calculated with Kimura’s 2 parameter model and substitutions included transitions and transversions. Gaps and missing data were treated as pairwise deletions. Bootstrap confidence values were calculated from 100 bootstrap replications. From this distance matrix, principal coordinate analysis (PCoA) was also carried out using GenAlEx [18] with standardization.

## Results

### PCA based ancestry estimation

**Fig 1:**
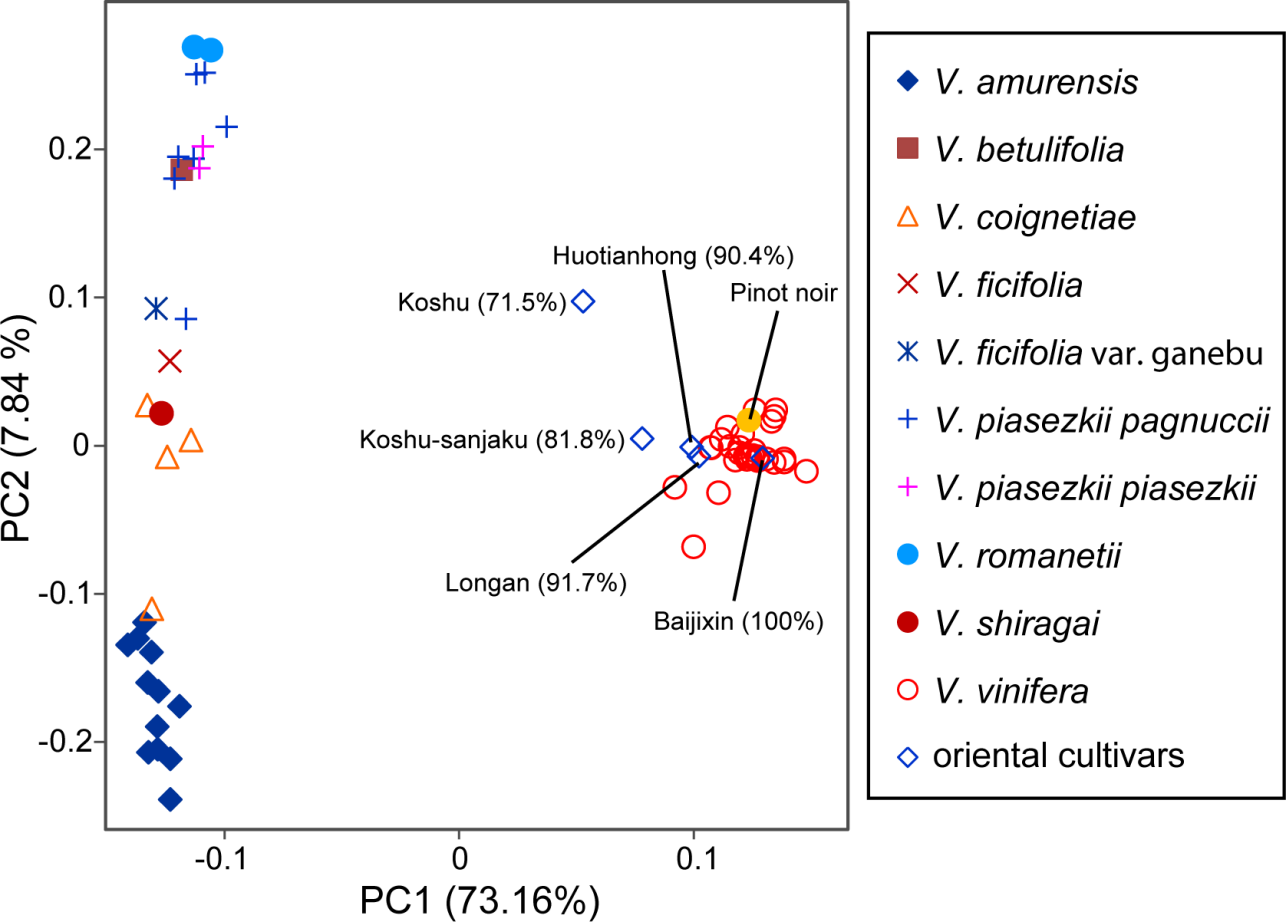
PCA plot of six genotyped grapevines projected on to principal components calculated from 33 accessions of East-Asian wild *Vitis* species and 33 cultivars of *V*. *vinifera*. Numbers in parentheses are estimated *V*. *vinifera* ancestry proportions based on PC1.

PCA of the two ancestral populations, wild East Asian species and *V*. *vinifera*, resulted in a clear separation of these two groups along PC1. Samples from the four East Asian wild species (*V*. *amurensis*, *V*. *coignetiae*, *V*. *ficifolia var*. *ganebu*, and *V*. *shiragai*) genotyped as part of this study all clustered with the East Asian wild ancestral population genotyped in [14]. The Pinot Noir sample genotyped as part of this study also clustered as expected with the 33 *V*. *vinifera* cultivars genotyped in [15] and had an estimated *V*. *vinifera* ancestry proportion of 1.0. The *V*. *vinifera* ancestry proportions for the five East Asian cultivars genotyped as part of this study had values ranging from 0.715 to 1 (Fig 1).

### Analysis of chloroplast diversity

We find that the partial chloroplast sequence of Chardonnay is identical to the reported sequence of *V*. *vinifera* cv. Maxxa (NC_007957.1) except for one site at position 205, as reported by Tabidze et al [19]. Differences were observed between Koshu and Chardonnay at 8 sites (Table 1). All other oriental cultivars share an identical chloroplast sequence with Chardonnay (*V*. *vinifera*).

**Table 1:** Differences in partial sequence of chloroplast DNA between cultivars with *V*. *vinifera* type chloroplasts and cultivars with non-*V*. *vinifera* type chloroplasts. Nucleotide positions of Maxxa (NC_007957.1) are shown as the reference.

Region	*trnH-psbA*		*trnK-rps16*	*trnF-ndhJ*	*rpl32-trnL*			
nucleotide position	260	301	4942	52400–52403	119887	119996–120004	120130–120139	120242
Charodonnay, Longan, Huotianhong, Baijixin, Koshu-sanjaku	T-	G	C	CATA	C	TTTTTTTTT	GGAAACAGAA	G
Koshu	TC	A	A	-	T	TTTTTTTT	-	T

**Fig 2:**
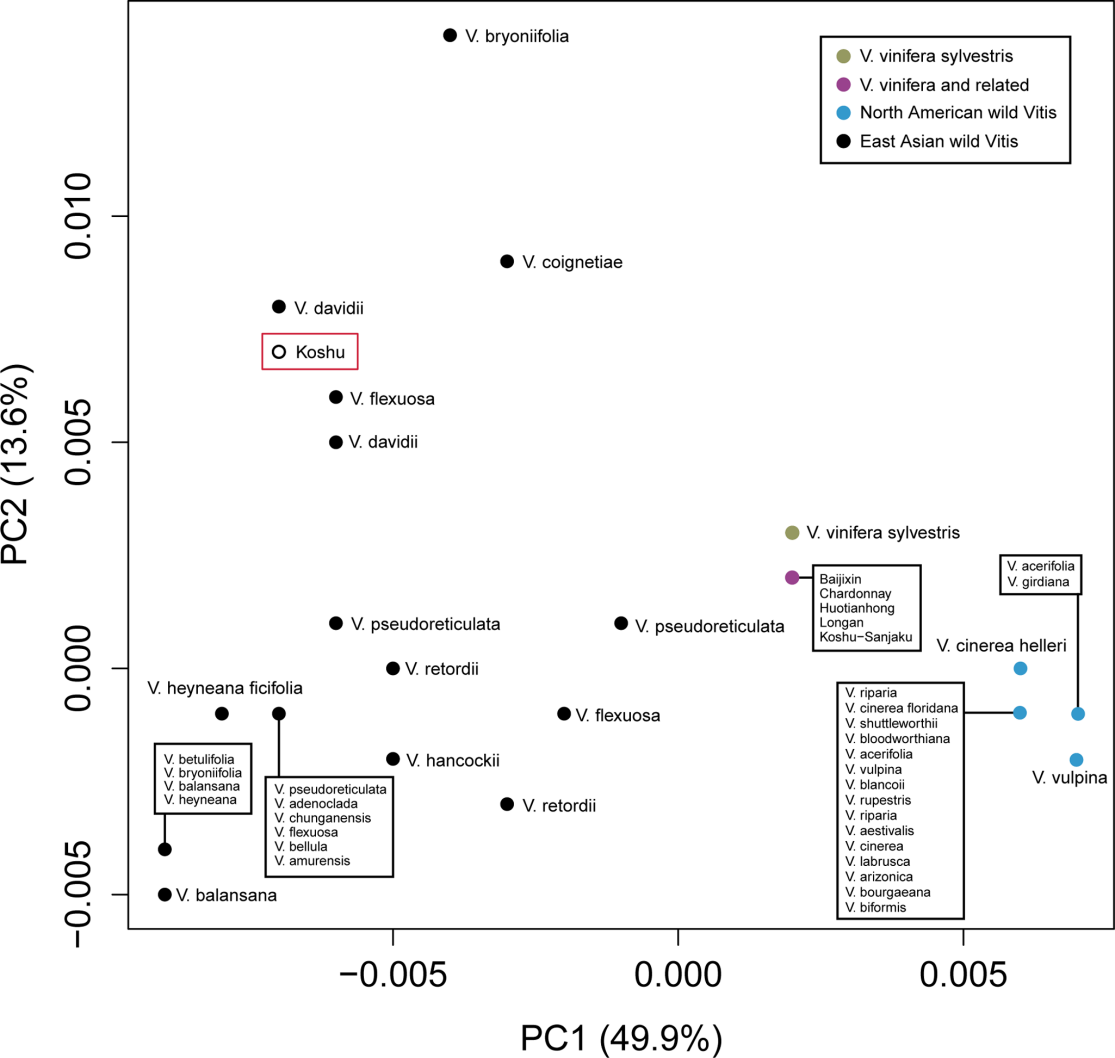
Principal coordinates analysis plot based on partial chloroplast sequences from diverse *Vitis* accessions. The proportion of variation explained by each PC is found in parantheses along each axis.

A PCoA plot based on a chloroplast genetic distance matrix enables the relationships among the accessions’ chloroplast sequences to be visualized in two dimensions (Fig 2). Fig 2 shows that Koshu is closest to the Asian wild species *V*. *davidii* and *V*. *flexuosa*. Because all other oriental cultivars share an identical chloroplast sequence with Chardonnay, they are represented in the plot as a single point. A neighbor-joining tree also supports the notion that Koshu derives its chloroplast DNA from an Asian wild species, as it clusters most closely with *V*. *davidii* (S1 Fig).

## Discussion

The ancestry of some of the most common East Asian grape cultivars is often claimed to be derived exclusively from the domesticated grape, *V*. *vinifera*, which originated in the Caucasus region of Central Asia. Studies investigating these claims have so far been limited in marker number and/or sample size. Here we use a set of genome-wide ancestry informative SNPs and chloroplast DNA sequences to investigate the potential contribution of wild grape species to the genomes of present-day East Asian grape cultivars.

Accessions of wild Asian grape species (*V*. *amurensis*, *V*. *coignetiae*, *V*. *ficifolia var*. *ganebu*, and *V*. *shiragai*) genotyped for the present study clustered closely in PC space with wild Asian species genotyped in a previous study using the Vitis9KSNP array [14] (Fig 1). In addition, the *V*. *vinifera* accession we genotyped here as a control (Pinot Noir) also clustered with the *V*. *vinifera* accessions genotyped previously [15]. Thus, we are confident that the genotype data obtained from the AIMs we identified are concordant with genotype data generated from the Vitis9KSNP array and that the use of merged data for the purposes of ancestry assignments is justified.

Our analysis of 48 AIMs in the Japanese grapes Koshu and Koshu-sanjaku suggests that 20–30% of their genomes are derived from wild *Vitis* species (Fig 1). Thus, we conclude that Koshu and Koshu-sanjaku are not cultivars of *V*. *vinifera*, in contradiction to previous work [3, 7–9]. It is likely that hybridization with wild *Vitis*, intentional or by chance, occurred at some point in the history of these commercially successful Japanese wine grapes. We find that the chloroplast genome of Koshu appears most similar to *V*. *davidii* and not *V*. *vinifera* (Fig 2 and S1 Fig), indicating that at least one of its East Asian ancestors was a female plant.

Although we can safely conclude that Koshu is an interspecific hybrid, we cannot conclusively identify the type or number of wild species in its lineage as these species may be extinct, unidentified, or simply missing from the present analysis.

It is also worth noting that, based on our analysis of partial chloroplast DNA sequences, accessions from a single *Vitis* species did not always cluster together in genetic space: it was often the case that accessions from a single species were more genetically distant than accessions from different species (Fig 2 and S1 Fig). This may be due to curation error, or it may the case that there is incomplete lineage sorting: the partial chloroplast sequences studied here may segregate within and among *Vitis* species. The clear separation of North American and Asian wild *Vitis* species, together with the close relationship between *V*. *vinifera* and its wild ancestor *V*. *vinifera subsp*. *sylvestris*, provide support that the genetic distances based on chloroplast DNA sequences at least partially capture the evolutionary relationships among the samples studied here (Fig 2 and S1 Fig). A more comprehensive analysis of chloroplast DNA sequences is required to refine the relationship between Koshu and its chloroplast ancestor.

The errors associated with the PCA-derived ancestry estimates presented here provide support to our conclusions and refine the possibilities of how wild introgression took place in the history of commercial East Asian grapes. Using simulated offspring from F1 and F2 hybrids between *V*. *vinifera* and wild *Vitis* species, Sawler et al. [12] used the same PCA-based approach to accurately estimate the simulated offsprings’ *V*. *vinifera* ancestry with 95% confidence intervals of approximately ± 0.04. Assuming similar variation in estimates in the present study, Koshu (71.5% *V*. *vinifera*) could therefore be F2 plants derived from an F1 hybrid parent (*V*. *vinifera* x East Asian wild species) and a *V*. *vinifera* parent. We cannot rule out the possibility, however, that this cultivar has a more complex pedigree including wild Asian *Vitis* and *V*. *vinifera*. Koshu-sanjaku (81.8% *V*. *vinifera*) has a higher estimated proportion of *V*. *vinifera* ancestry than Koshu and, according to the estimated error, is unlikely to be an F2 hybrid as described above. Thus, Koshu-sanjaku is more likely to be the result of a complex pedigree with *V*. *vinifera* and East Asian wild *Vitis* relatives. Longan (91.7% *V*. *vinifera*) and Huotianhong (90.4% *V*. *vinifera*) both cluster close to the *V*. *vinifera* population in Fig 1. They may be genetically distinct *V*. *vinifera* cultivars or possess a very small proportion of wild *Vitis* ancestry. Finally, we determined that the Chinese cultivar Baijixin (100% *V*. *vinifera*) is in fact most likely a *V*. *vinifera* cultivar. With sufficiently dense genotype data from a comprehensive sample of *V*. *vinifera* and oriental cultivars, it may be possible in the future to identify the *V*. *vinifera* cultivars that contributed to the ancestry of commercial oriental cultivars.

Taking into account the results of the SNP based ancestry estimation and observed genetic relatedness to wild *Vitis* based on chloroplast sequencing, the simplest hypothetical origin of Koshu is that it originated from a cross between an F1 hybrid (*V*. *davidii* or related x *V*. *vinifera*) and a second *V*. *vinifera* cultivar. *V*. *davidii* is a synonym of *V*. *armata*, and has a wide geographic distribution in China [20]. Cultivars of *V*. *davidii* are grown in China, and are used for both wine making as well as breeding stock. The branches of *V*. *davidii* possess many spines, and it is sometimes referred to as “spiny *Vitis*”. The young shoots of Koshu have small spines that may be a result of *V*. *davidii* ancestry. In addition, *V*. *davidii* is highly resistant to disease and is tolerant to wet climates [20]. These traits may have contributed to the success of Koshu given the wet and hot summers in Japan. However, nearly all anthocyanins of *V*. *davidii* are delphinidin-based and diglucosides [21], which differ from those produced by Koshu. As previously discussed, *V*. *davidii* is only one of many possible ancestors of Koshu, and further work examining more samples and markers will be required to confirm or refute the conjectures outlined here.

Studies of European grape cultivars have demonstrated introgression from local wild vines as humans brought grapes into Europe [15]. While our data do not enable us to determine the timing of the hybridization events that led to the East Asian cultivars studied here, there was likely ample opportunity for wild introgression during the journey of the domesticated grape along the Silk Road trade route to Japan. Further studies are required to refine the timing and precise nature of the hybridization events that led to the grapes currently grown commercially in East Asia.

## Supporting Information

S1 Fig NJ tree based on partial sequence of chloroplast DNA. Labels on the branches are bootstrap confidence values. The letters following the species names refer to the IDs used in [16].
(TIF)

S1 Table Primer sequence for the 48 ancestry informative SNPs genotyped. The SNP ID is composed of the chromosome, followed after the colon by the position according to the 8x Pinot Noir (PN40024) genome sequence [13].
(XLSX)

S2 Table SNPs of 10 grape accessions for 48 AIMs. The SNP ID is composed of the chromosome, followed after the colon by the position according to the 8x Pinot Noir (PN40024) genome sequence [13]. When only a single allele appears, it indicates that the sample is homozygous for that allele.
(XLSX)

S3 Table Primers for partial sequencing of chloroplast DNA. (XLSX)
