# Supplementary figures and images for: Genetic Analysis of East Asian Grape Cultivars Suggests Hybridization with Wild Vitis
Source: PLoS One. 2015 Oct 21;10(10):e0140841. doi: 10.1371/journal.pone.0140841 (PMC4619069; doi:10.1371/journal.pone.0140841)

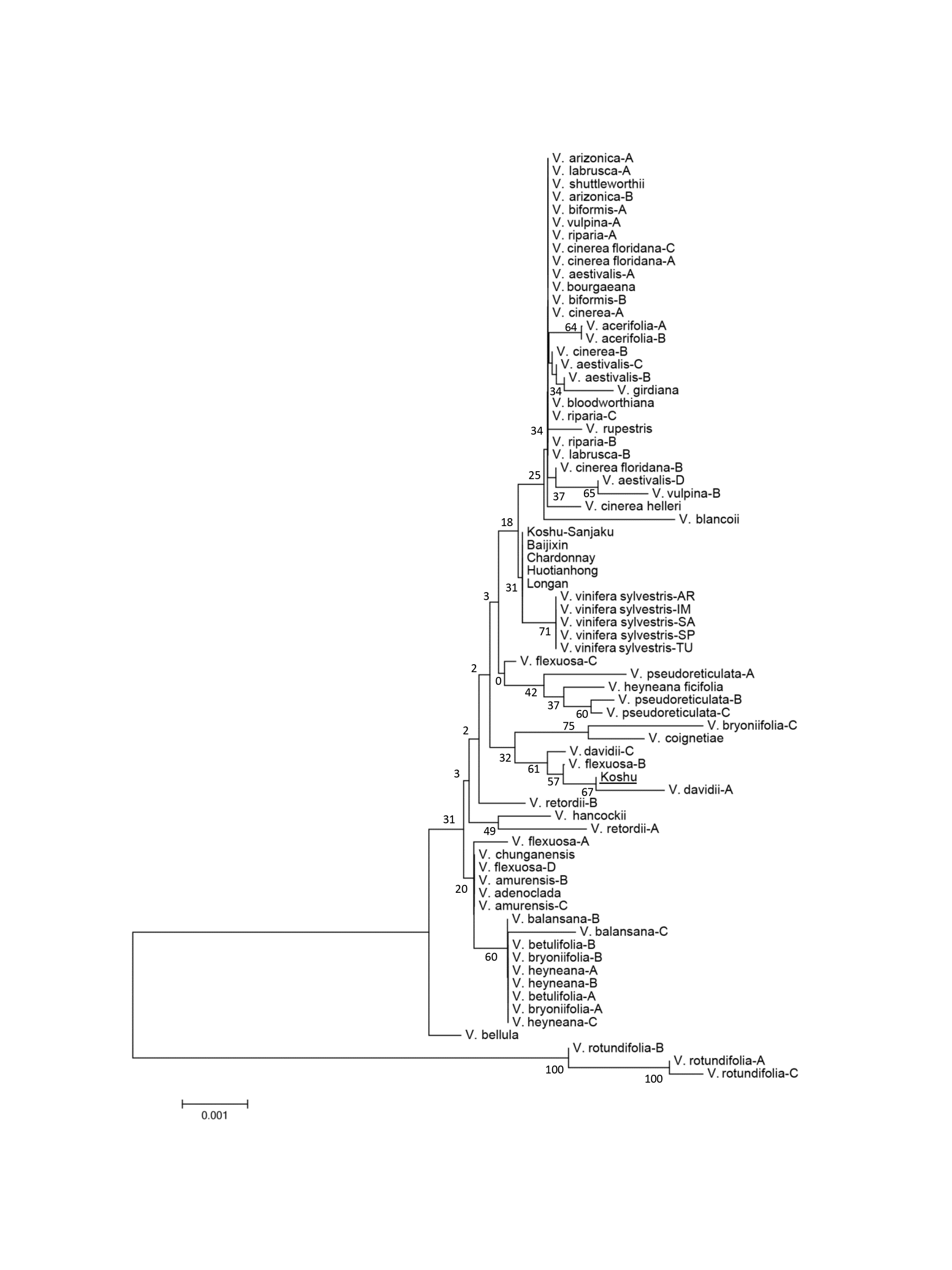

Supplement: S1 Fig — Labels on the branches are bootstrap confidence values. The letters following the species names refer to the IDs used in [16]. (TIF) [file pone.0140841.s001.tif]
